# Web-Based Presence for Social Connectedness in Long-Term Care: Protocol for a Qualitative Multimethods Study

**DOI:** 10.2196/50137

**Published:** 2023-10-27

**Authors:** Anna Garnett, Halyna Yurkiv, Richard Booth, Denise Connelly, Lorie Donelle

**Affiliations:** 1 Arthur Labatt Family School of Nursing University of Western Ontario London, ON Canada; 2 School of Physical Therapy University of Western Ontario London, ON Canada; 3 University of South Carolina Columbia, SC United States

**Keywords:** social connectedness, web-based presence technology, long-term care homes, older adults, research protocol, mobile devices, social interaction, social interactions, connectedness, lonely, loneliness, older adult, gerontology, ageing, geriatric, geriatrics, elder, elderly, nursing home, assisted living, web-based presence, conceptual, isolation, mobile phone

## Abstract

**Background:**

The COVID-19 pandemic and resultant restrictions on social gatherings significantly impacted many peoples’ sense of social connectedness, defined as an individual’s subjective sense of having close relationships with others. Older adults living in long-term care homes (LTCHs) experienced extreme restrictions on social gatherings, which negatively impacted their physical and mental health as well as the health and well-being of their family caregivers. Their experiences highlighted the need to reconceptualize social connectedness. In particular, the pandemic highlighted the need to explore novel ways to attain fulfilling relationships with others in the absence of physical gatherings such as through the use of a hybridized system of web-based and in-person presence.

**Objective:**

Given the potential benefits and challenges of web-based presence technology within LTCHs, the proposed research objectives are to (1) explore experiences regarding the use of web-based presence technology (WPT) in support of social connectedness between older adults in LTCHs and their family members, and (2) identify the contextual factors that must be addressed for successful WPT implementation within LTCHs.

**Methods:**

This study will take place in south western Ontario, Canada, and be guided by a qualitative multimethod research design conducted in three stages: (1) qualitive description with in-depth qualitative interviews guided by the Technology Acceptance Model (TAM) and analyzed using content analysis; (2) qualitative description and document analysis methodologies, informed by content and thematic analysis methods; and (3) explicit between-methods triangulation of study findings from stages 1 and 2, interpretation of findings and development of a guiding framework for technology implementation within LTCHs. Using a purposeful, maximum variation sampling approach, stage 1 will involve recruiting approximately 45 participants comprising a range of older adults, family members (30 participants) and staff (15 participants) within several LTCH settings. In stage 2, theoretical sampling will be used to recruit key LTCH stakeholders (directors, administrators, and IT support). In stage 3, the findings from stages 1 and 2 will be triangulated and interpreted to develop a working framework for WPT usage within LTCHs.

**Results:**

Data collection will begin in fall 2023. The findings emerging from this study will provide insights and understanding about how the factors, barriers, and facilitators to embedding and spreading WPT in LTCHs may benefit or negatively impact older adults in LTCHs, family caregivers, and staff and administrators of LTCHs.

**Conclusions:**

The results of this research study will provide a greater understanding of potential approaches that could be used to successfully integrate WPTs in LTCHs. Additionally, benefits as well as challenges for older adults in LTCHs, family caregivers, and staff and administrators of LTCHs will be identified. These findings will help increase knowledge and understanding of how WPT may be used to support social connectedness between older adults in LTCHs and their family members.

**International Registered Report Identifier (IRRID):**

PRR1-10.2196/50137

## Introduction

### The Impact of Restrictions on Social Connectedness

The COVID-19 pandemic and resultant restrictions placed upon Canadians and residents of many other countries (ie, stay-at-home orders and social distancing mandates) significantly impacted elements of many people’s social connections over the last 2 years [1,2]. *Social connectedness*, defined as an individual’s subjective sense of having close relationships with others [3,4] is necessary for health and well-being [5]. During multiple phases of the pandemic, social connectedness was severely compromised for many people both directly and indirectly by restrictions placed on face-to-face engagements [6]. In Canada, older adults in congregate or institutional living environments, such as long-term care homes (LTCHs), were greatly affected during these times [7]. Older adults in LTCHs experienced some of the highest rates of COVID-19 mortality and morbidity of any population group [8,9]. Along with the systematic failure of some LTCH facilities [10], almost all LTCHs were mandated to implement extreme restrictions on visitors and staff, as an emergency infection control [11]. These drastic measures increased isolation and decreased the emotional well-being of older adults in LTCHs [6,12,13].

### Progression of Social Isolation Impact on Older Adults

Before the COVID-19 pandemic, older adults in LTCHs were at risk of experiencing social isolation due to a variety of factors such as challenges with building new relationships, decreased autonomy, and geographical constraints on maintaining face-to-face or in-person connections with friends and family [14]. In older adults, social isolation is associated with negative health impacts such as reduced motor function, depression, cognitive decline, and mortality [15-19]. Similarly, loneliness is also implicated in health declines [20]. As people age, their likelihood of experiencing social isolation and loneliness increases [21,22]. In the United States, upwards of 40% of people older than 60 years reported feeling lonely [23]. In Canada, older adults also report elevated rates of loneliness and social isolation [24]. Moving to LTCHs can increase older adults’ likelihood of being lonely or socially isolated with reported rates of loneliness in older adults living in LTCHs varying from 22% to 75% [20,25,26].

With the start of the pandemic, social connectedness among older adults within LTCHs was severely compromised both directly and indirectly by restrictions placed upon visitors and other infection control measures [27]. This physical isolation of older adults in their rooms thereby compounded any preexisting impacts of isolation [6]. Detrimental impacts on older adults’ mental health associated with quarantine measures include emotional disturbance, insomnia, and low mood [28,29]. While these visitor restrictions were implemented in good faith as an effort to mitigate the spread of COVID-19, many older adults during the height of the pandemic died alone, with little to no connection with their family members or friends [30].

In the initial stages of the COVID-19 pandemic, research findings suggested that older adults may be less vulnerable to detrimental mental health effects of the disease and associated health measures, than their younger peers [30,31]. However, with time, additional studies indicated that the impacts of COVID-related restrictions and the resultant social isolation will have long-lasting repercussions on older adults’ health and well-being [27]. Furthermore, the protracted restrictions associated with the ongoing nature of the pandemic continue to negatively impact the physical and mental health of older adults and their family caregivers [9,12,30]. In Canada, during the first wave of the COVID-19 pandemic, when families were prohibited from in-person visits, there were 7260 deaths of older adults across LTCHs and retirement homes, representing 79% of all COVID-19 deaths [7]. Consequently, during the height of the pandemic, many older adults died isolated and alone [30-33]. Since that time, the policy changed first resulting in modified visitor restrictions that allow family members to be together in palliative circumstances and then removing visitor restrictions altogether [34]. However, challenges relating to the social isolation of older adults within LTCHs remain.

As COVID-19 led to widespread limitations on in-person gatherings and for a time halted in-person visits to LTCHs, attention turned to technology as a means to communicate and facilitate social connectivity between family members and older adults [35]. As we move forward from the pandemic it is critical to develop robust systems now, to mitigate the harmful unintended side effects (eg, decreased social connectedness and well-being) of infection control policies.

### Web-Based Presence Technology

Web-based presence is the use of networked digital telecommunications technology to create the impression that a user is present in another environment [36]. Digital apps (eg, Zoom [Zoom Video Communications] and FaceTime [Apple Inc]) provide web-based face-to-face connection and may support social connectedness by providing meaningful social engagement in addition to being a point of access to community environments for older adults [37]. For LTCHs, web-based presence technology (WPT) has the potential to support social connectedness as a supplement to in-person engagement or a stand-in during outbreak situations such as the current COVID-19 pandemic. Acknowledgement of this potential has been highlighted by the COVID-19 pandemic, which has been a catalyst for myriad and lasting social changes, including the vastly accelerated use of WPT for communication in people’s personal and professional lives. However, the general uptake of WPT across LTCHs has lagged behind other sectors [38] for reasons including age-related bias about older adults’ physical and cognitive ability to use technology, costs of equipment, and a lack of education and support of staff, family, and older adults to use technology devices such as tablets or other mobile devices [39].

Early efforts to explore the application of technology within LTCH settings focused on aspects such as resident safety. However more recently, the role of technology to support social connectedness is also being investigated [40]. Emerging findings suggest that challenges of implementing WPT in LTCHs include limitations in staff shortages, training for staff on the use of WPT, dated LTCH building infrastructure, funding to support additional staffing and technology equipment, and processes supporting consistent use of WPT [41]. Addressing these challenges entails more than just the provision of additional funding. Instead, it asks for an enhanced systemic and context-specific understanding of technology implementation [42]. This understanding has to be cocreated with all relevant LTCH stakeholders (eg, residents, family members, staff, and administration) to effectively address user needs as well as financial, operational, and administrative constraints.

WPT can provide family members of older adults in LTCHs with the opportunity to visually observe the well-being of the older adult in real time. Visual confirmation of these older adults’ well-being can contribute to family members’ trust and confidence in the care being provided by LTCH staff [43]. Admittedly, WPT is not a panacea for fostering social connectedness among older adults in LTCHs [44]. However, it may be profoundly important to supplement in-person activities with *hybridized web-based and in-person approaches* for social connectedness in the context of distancing measures or other constraints on in-person engagement.

### Objectives

Given the potential benefits of WPT within LTCHs and the need to understand associated shortcomings, the proposed research goal is to explore experiences regarding the use of WPT in support of social connectedness between older adults in LTCHs and their family members, and to identify the contextual factors that must be addressed for successful WPT implementation in LTCHs. This study has 3 aims. First, to explore how older adults in LTCHs, their family members, and LTCH staff members experience, benefit from, and envision the role of WPT to support social connectedness in LTCHs. Second, to interview key stakeholders such as managers and directors of LTCHs, and to examine LTCH policy, funding, management, and operations in order to identify institutional challenges and facilitators impacting the uptake and spread of WPT within LTCHs. Third, to triangulate the findings from stages 1 and 2 to develop a framework that can inform the formal implementation of WPT in support of social connectedness within LTCH**.**

## Methods

### Study Team

The faculty team recognizes that there is an implicit bias against some groups’ progression to academia’s higher ranks. Our faculty team is deeply committed to equity and diversity in recruiting, hiring, and training students. This research team will comprise emergent and seasoned scholars and researchers and a research assistant who is a graduate student at the university where the principal investigator is employed.

### Approach

#### Overview

This study will use a qualitative multimethod research design conducted in 3 stages. The multimethod design will be informed by the Technology Acceptance Model (TAM) and entail the use of qualitative description using content analysis (stage 1) and thematic and document analysis methodologies (stage 2). The overarching objective will be to triangulate the findings from stages 1 and 2 to create a working framework to inform the implementation of WPT in LTCHs. Stage 1 is focused on data collection through in-depth interviews with stakeholders (older adults living in LTCHs, their family members and LTCH staff) to inform an initial understanding of the perceived usefulness, ease of use, challenges, attitude toward, and human resource availability to support social connectedness using WPT. Stage 2 will involve the examination of relevant documents (eg, information technology and operational policy documents, budgetary documents and training manuals) and in-depth interviews with key LTCH administrators such as directors and managers as well as salient staff (eg, information technology) that will describe the LTCH environment, its functioning, and regulations to develop an understanding of the institutional factors that have a bearing on potential web-based presence implementation. In stage 3, the data from stages 1 and 2 will be used to triangulate the findings and interpret them to develop a working framework for WPT implementation within LTCHs.

#### Stage 1: Interviews and Framework Development

##### Design

The first stage of this study will use qualitative description to explore how older adults in LTCHs, their family members, and LTCH staff experience and understand the use of WPT in support of the social connectedness of older adults in LTCHs [45]. The outcome of this exploratory enquiry will be combined with the outcome of stage 2.

##### Ethical Considerations

As the initial step of stage 1, ethics approval for the proposed study will be sought from the applicable research ethics boards (Western University Health Science Research Ethics Board). Once ethics approval has been obtained, data collection will be initiated. The research team anticipates beginning the participant recruitment phase in late autumn 2023.

The principal investigator will manage all data generated with this research to ensure effective data use and security. Standardized data file naming, storage (including metadata) and backup procedures will support data sharing and protect the data against catastrophic loss. Data will be stored on a password-protected university server to protect against unauthorized access, in keeping with research ethics board requirements (ID#2023-121993-78969).

##### Participants

To conduct the interviews in stage 1, we will use purposeful and maximum variation sampling to recruit a range of older adults, family members (30 participants) and staff (15 participants) within several LTCH settings with a total recruitment goal of approximately 45 participants [46]. A review of the literature on sample size across 55 qualitative descriptive studies found that about half of studies (24 studies) had a sample size ranging from 8 to 20 participants and approximately 15% (8 studies) of included studies had a sample size of up to 50 participants [46]. Therefore, a total sample size of 45 for stage 1 is deemed to be congruent with other research conducted using a qualitative descriptive method. Data collection and analysis will take place simultaneously and will continue until the data provides a rich description of the participants’ experience in using or supporting the use of WPT for the purposes of social connection. It is expected that data collection will conclude once data saturation has been obtained across the range of participants [47]. Participants will be recruited from 3 LTCHs (anonymized). Given the goal of gaining a holistic understanding of the role of WPT implementation in LTCHs, we hope to recruit a broad range of participants (older adults, family members and staff including, registered practical nurses, registered nurses, personal support workers, and recreation therapists).

Inclusion criteria for this stage will be English speaking and the ability to participate in a one-on-one interview using Zoom technology. Potential older adult participants with mild cognitive decline living in LTCHs or receiving outpatient care will be assessed for suitability to participate in this study by LTCH staff first before any study information will be shared with them. Exclusion criteria applied will be the inability to converse in English and advanced cognitive decline.

##### Screening Process

The screening process will be conducted by partnering organizational staff. As part of LTCH guidelines a regular Montreal Cognitive Assessment screening [48] is used to assess residents’ cognitive dimensions such as language, memory, visual and spatial thinking, reasoning, and orientation status. Upon receiving study information, partnering staff will identify potentially suitable participants and share this study’s information with them on behalf of the researchers.

##### Setting

Individual in-depth interviews with older adults, their family members, and LTCH staff will take place in-person, via phone, or Zoom call depending on participant preferences and applicable COVID-19 restrictions at an agreed-upon date and time.

After the screening and deeming an older adult eligible to participate in this study, a research assistant will connect with a recreational therapy department staff or a caregiver in order to assist older adult participants with setting a study interview appointment, securing a quiet room, accessing an electronic device at their LTCHs, and helping them join a Zoom meeting to participate in an interview at an agreed upon date and time.

##### Data Collection

We will conduct semistructured in-depth interviews with a range of older adults, family members, and staff in several LTCHs. The purpose of conducting these interviews will be to explore participants’ perceptions of how WPT has been used and can be used, and to identify potential barriers to supporting social connectedness between older adults and family members within LTCH settings using WPT. Interview questions will be informed by extended the TAMs as widely applied to questions of health technology implementation [49-51]. The TAM and its extensions are based on 2 belief constructs: perceived technology usefulness and perceived ease of technology use [50,51]. Building on these constructs, a range of interview questions tailored for type of participant (staff, older adult living in LTCHs, and family member) will be posed to participants (eg, can you describe your experience with using technology such as smartphones or tablets for speaking with your family). For a full list of interview questions please see Multimedia Appendix 1. In addition to in-depth interviews, observational field notes will be kept throughout this study thereby enhancing the data set [52].

##### Data Analysis

Data will be analyzed using directed content analysis, a qualitative analytic technique characterized by a structured stepwise process of coding the data and organizing it into categories or themes [53]. The goal of the analysis will be to provide the initial data for a framework to guide web-based presence implementation in LTCHs. As such, the key constructs of the TAM, perceived usefulness and ease of use of technology, will serve as initial codes to guide analysis. Data that do not lie within one of the key codes will be labeled with a new code and organized into new categories or organized depending on their suitability as subcategories under the initial codes [53]. Throughout the entire sampling and data collection process, data analysis will be conducted in a concurrent and iterative process. Interviews with participants (in-person, via phone or Zoom depending on participant preferences and applicable COVID-19 restrictions) will be audio recorded, transcribed verbatim prior to analysis. Study rigor will be ensured through member checking, verifying data by contacting a subset of participants a second time to verify their intended meaning [54]. In addition, those engaged in the research process will practice reflexivity, acknowledging biases they may introduce to the research, and other strategies such as ethical conduct and ensuring the trustworthiness (eg, confirmability of data through triangulation between interviews and field notes) of this study data will be used [55].

#### Stage 2: Interviews With Key Stakeholders and Document Analysis

##### Design

To further develop insights related to the challenges and facilitators impacting the spread of WPT within LTCHs, stage 2 will consist of 2 elements: interviews with stakeholders using a qualitative descriptive approach, and document analysis. The in-depth interviews will be analyzed using conventional content analysis, an analytic technique that will facilitate increased understanding in the absence of guiding theory [53]. Document analysis, a systematic process of evaluating documents, is often used in conjunction with other qualitative methods in order to facilitate triangulation. Including a wide variety of documents (eg, policy documents, correspondence, and institutional reports), document analysis will be used as a means to enhance this study’s findings with its potential to provide greater insight, information, and understanding [56].

##### Participants and Setting

Approximately 10-15 stakeholder participants will be recruited using convenience sampling. Given the focused nature of the interviews in stage 2, it is expected that fewer participants will be required to reach data saturation (approximately 5 to 8 per participating LTCHs). Participants will be invited to participate through targeted outreach via the (anonymous) Long-term Care Associations and through current relationships with LTCHs and professional nursing organizations. Key stakeholders will involve a range of functions or positions including directors of care, chief executive officers, director of community connections, and director of information technology innovation. Interviews with key stakeholders will take place in person, via phone or Zoom call depending on participant preferences and applicable COVID-19 restrictions at an agreed-upon date and time.

##### Data Collection

A qualitative description will be the methodological approach used to conduct in-depth interviews with key stakeholders (eg, IT personnel and LTCH management) [45,53]. Purposeful sampling techniques such as theoretical sampling are commonly used to recruit individuals or focus groups and data is often analyzed using content analysis when using a qualitative description approach [46]. Prior research in health technology implementation has stressed the importance of the role that system factors play in successful technological implementation in clinical settings [57]. Insights gained during stage 1 (eg, identification of barriers to the use of WPT) will inform the development of an interview guide targeted at increasing understanding of how potential modifications in structural and institutional factors could facilitate the spread of WPT in LTCHs.

Contributing documents to the document analysis component of this stage of this study will include annual LTCH budget reports, annual reports, strategic plans, LTCH funding policy documents, and regulatory documents as well as relevant communication documents (anonymous). These documents can potentially add information that can inform understanding of WPT use in LTCHs such as resource and budgetary allocation for technology and support personnel.

##### Data Analysis

In-depth interviews with participants will be audio recorded, transcribed verbatim, and analyzed using conventional qualitative content analysis with codes emerging through an iterative process of data collection and analysis [53]. Identified key documents will be analyzed using *conventional content analysis.* This will be an iterative process of reading the interviews, identifying initial coding categories based on the results from the interviews with key stakeholders and then organizing the codes into categories [53].

Analysis of documents will entail an iterative process which includes scanning documents, preliminary reading in detail, and finally organizing the data using strategies such as coding and categorizing the data. Concomitant to the analysis will be ensuring that the documents are credible, accurate, and able to inform the research questions [56].

#### Stage 3: Explicit Between-Methods Triangulation and Conceptual Framework Development (Methods)

In the concluding stage, there will be *explicit between-methods triangulation* of the findings of the respective methodological approaches (qualitative description using directed content analysis and conventional content analysis and document analysis) used in stages 1 and 2 to develop a final conceptual framework [58,59]. Findings from the various methodological approaches used in stages 1 and 2 will be systematically gathered and sorted to allow discerning patterns of overlapping and complementing evidence to establish the emerging final conceptual framework [60].

## Results

The result of this iterative analytic process will inform theoretical understanding of how older adults in LTCHs, their family members and staff experience and envision the application of WPT to support social connectedness. After stage 1, a total of 45 participants will have completed in-depth interviews and their data will have been used to generate theory related to how WPT can be used within LTCHs to support social connectivity. The findings emerging from stage 1 will be used to directly inform stage 2’s data collection as well as the overall development of a conceptual framework (stage 3).

Findings emerging from key stakeholders' interviews as well as document analysis will be used to inform *explicit between-methods triangulation* in stage 3 by expanding on findings through the inclusion of data obtained through other methods [61]. Expected contributions from the document will include a greater understanding of factors such as fiscal constraints, an organizational structure including staffing complement, and controlling regulatory and policy frameworks required for the successful implementation and spread of WPT for social connectedness within LTCHs.

The findings emerging from this study will provide deeper insights and understanding about the factors, barriers, and facilitators to embedding and spreading WPT in LTCHs and benefit older adults in LTCHs, family caregivers, and staff and administrators of LTCHs. Going forward, the conceptual framework will have utility to inform approaches to embedding web-based presence as a mainstreamed communication tool to support social connectedness among older adults in LTCHs and their family members.

## Discussion

### Principal Findings

A growing body of literature demonstrates the benefits of various communications technologies such as social networking apps on older adults’ well-being, independence, and sense of connectedness with friends and family [62]. Knowing that social connectedness is a key contributor to the well-being of older adults in LTCHs [2], it will be important to optimize communications web-based technology in an efficient and timely way to avoid compounding further unintended detrimental effects to limit transmission of COVID-19, or any other future pandemics or epidemics.

As society continues to experience evolving disruptions to the ability to engage with one another, there is a need to reconceptualize social connectedness and to identify novel ways to attain fulfilling relationships with others particularly in the context of LTCHs. In the context of LTCHs, this could be represented by a hybridized system of web-based and in-person presence. However, more evidence is required to determine whether older adults in LTCHs and their family members benefit from WPT (eg, smartphones and tablets) as an approach to support their social connectedness. Further, there is a need to better understand how WPT can be implemented in LTCHs.

While WPT may have the potential to support meaningful social connectedness among many older adults in LTCHs and their family members, to date numerous uncertainties remain regarding leveraging WPT for social connectedness. These uncertainties include (1) the effectiveness of using WPT for social connectedness by older adults with cognitive impairments, (2) best practices related to the use of WPT by older adults with cognitive impairment, and (3) the role of extra supports for older adults with cognitive impairment when using WPT [44]. Additionally, it will be important to identify how an older adults’ physical condition could impact their ability to use WPT. For example, arthritis, vision impairments or neurodegenerative conditions such as Parkinson disease could necessitate the need for adaptive technology or additional assistance [63,64].

### Limitations

The proposed multistage approach and methodological triangulation will require the engagement of multiple stakeholders. Potential limitations may include challenges associated with conducting interviews with older adults living in LTCHs who may have cognitive or physical conditions that could limit their participation in in-depth interviews. Additional support from family members, LTCH staff or researcher staff may be required to facilitate interviews with older adults. Researchers will use flexible approaches to interviewing including in-person and zoom interviews, accommodating interview times and an honorarium to recognize their time and contribution. Researchers will also use strategies that can support older adults’ participation such as providing documentation in larger font size and providing a list of questions ahead of time.

The data obtained through qualitative description will consist of subjective perceptions and recollections of using technology for social connectedness and therefore may be biased toward a particular type of experience. Direct user observations, attained using an ethnographic approach, might be able to produce data that have a lower likelihood of being biased toward memorable experiences. However, actualizing this approach within LTCHs would be very challenging to practically implement.

### Conclusions

The conceptual framework arising from this research on the role of WPT to support social connectedness between older adults in LTCHs and their family members will have a key role in informing LTCHs how to embed web-based presence as a mainstream communication tool to redefine and support social connectedness among older adults in LTCHs and their family members.

## Appendix

Multimedia Appendix 1 Interview guide.

## Abbreviations

LTCH: Long-term care home
TAM: Technology Acceptance Model
WPT: web-based presence technology

## Data Availability

Attached is the interview guide to be used in stage 1 of this study. See Multimedia Appendix 1.
