# Supplementary material for: Web-Based Presence for Social Connectedness in Long-Term Care: Protocol for a Qualitative Multimethods Study
Source: JMIR Res Protoc. 2023 Oct 27;12:e50137. doi: 10.2196/50137 (PMC10638636; doi:10.2196/50137)
Supplement: Multimedia Appendix 1 [file resprot_v12i1e50137_app1.docx]

Multimedia Appendix 1

Virtual Presence in Long-term Care

Staff Interview Guide

**Constructs to consider and keep in mind during interviews:**

1. Perceived usefulness
2. Perceived ease of use
3. Attitude to technology for social connectedness
4. Actual use of technology for social connectedness between older adults and family members

***Context***

- Can you describe your current role in LTC?
- Can you briefly describe how different types of technology are used in the LTC where you work.

***Focused Questions***

- Can you describe how technology use within the LTC where you work changed over the course of the COVID-19 pandemic?
- Did the use of technology change during COVID-19 and if so how would you describe the changes that took place?
- Can you describe an experience where you have used technology such as a tablet or smartphone to help an older adult living in long-term care to socially connect with a family member outside of the home?
  - Can you describe what worked well, what didn’t, why or why not, what hindered the experience?
  - Can you describe any additional technology/training or supports you would like to have in place to assist you in helping older adults and their
- Have you received any formal training to use technology such as tablets to help older adults living in long-term care to connect with their family members, and what did this consist of?
- Can you describe what would need in order to feel more comfortable in supporting older adults (OAs) with using virtual presence technology for social connection? *Prompt*: training, time, equipment
- Do you have a resource/technology support to refer to if you require assistance in using technology in any part of your job
- Can you describe your role in supporting older adults living in LTC to socially connect with their family either in person or using technology.
- Have you received formal training to support this role?
- Do you know if your LTC has formal policies that guide the use of technology for the purposes of social connection?
- Are there particular staff members that oversee technology use in the home?
- Can you speak to any situation where you had educational sessions regarding technology and virtual connectedness?
- Has your role in supporting social connectedness been accommodated in day-to-day role/schedule and if so can you explain how.

***Family Member Focus***

- Can you describe from your perspective how family members experienced using technology such as tablets and smart phones to socially connect with older adults living in LTC during COVID-19 and now?
- From your perspective what do you think family members or older adults living in LTC need to help them socially connect with one another using technology such as smart phones and tablets?
- Were family members and older adults using these technologies to socially connect prior to COVID-19?
- In your experience can you describe how prepared family members and older adults are/were to socially connect with one another using technology.
- Can you explain how you have supported family members in using technology to connect with an older adult living in LTC *prompts*: education, physically helping older adult etc.
- Has use of technology become normalized in LTC? Has this changed over the course of COVID-19?

***Next Steps***

- Going forwards what would you like to see to facilitate the use of technology for social connectedness within the LTC setting? *Prompts*: training, equipment, IT support
- Has your attitude about the use of technology to support social connectedness changed over the course of COVID-19 and if so how?
- Can you describe what is needed for optimal use of technology to support social connectedness within LTC.

Older Adult Interview Guide

**Constructs to consider and keep in mind during interviews:**

1. Perceived usefulness
2. Perceived ease of use
3. Attitude to technology for social connectedness
4. Actual use of technology for social connectedness between older adults and family members

***Context***

- Can you briefly tell me a bit about yourself e.g. how long you have lived here, what activities you like to do, nearby family members

***Focused Questions***

- Can you tell me about a time that you have used technology like smart phones or tablets to speak to your family when they can’t come into the LTC?
  - Can you describe what worked well, what didn’t, why or why not, what hindered the experience?
  - Can you describe any additional technology/training or supports you would like to have in place to assist you in helping older adults and their
- Can you describe what this experience (using technology) was like for you?
- Can you describe what type of help you received from staff members in the home to help you speak to your family using smart phones or tablets.
- Do you have your own smart phone or tablet or was one provided for you by the home or your family?
- Can you describe your experience with using technology such as smart phones or tablets for speaking with your family. *Prompts*: is this something you do regularly?
- Can you describe whether your use of technology has changed since the start of the COVID-19 pandemic? Prompts: were you using smart phones and tablets to connect before COVID-19? Do you still regularly use technology now that your family can regularly visit in person?
- Have you received any training in using technology such as smart phones or tablets since you have been here?
- Has your attitude towards using technology like smart phones and tablets changed since the COVID-19 pandemic? If so can you describe how?
- Are you aware of any programs or activities in the home that help you to use technology?
- Can you describe if and how using technology such as smart phones and tablets helped you feel more socially connected with your family when they couldn’t visit in person.
- Can you describe some challenges or difficulties you experienced when using technology such as smart phones or tablets to connect with your family.

***Next Steps***

- Going forwards what would you like to see to more use of technology to support connecting with family members?
- Can you explain what would help you to use technology for social connection.
- Can you describe what the LTC could do to help you use technology for social connection.
- Do you think you will use technology more going forwards? If yes or no, why or why not?
- Has your attitude about the use of technology to support social connectedness changed over the course of COVID-19 and if so how?

Family Member Interview Guide

**Constructs to consider and keep in mind during interviews:**

1. Perceived usefulness
2. Perceived ease of use
3. Attitude to technology for social connectedness
4. Actual use of technology for social connectedness between older adults and family members

***Context***

- Can you tell me a little bit about yourself and your relationship to the person you socially connect with in LTC
- Can you briefly describe how often you socially connect with your family member living in LTC (either in person or using technology) – can be now or during COVID-19

***Focused Questions***

- Can you describe how you use technology such as smart phones or tablets to connect with an older adult living in LTC?
  - Can you describe what worked well, what didn’t, why or why not, what hindered the experience?
  - Can you describe any additional technology/training or supports you would like to have in place to assist you in helping older adults and their
- Has your use of technology for social connecting with your family member living in LTC changed over the course of the COVID-19 pandemic? If so how would you describe the changes that took place? Prompts: were you using smart phones and tablets to connect before COVID-19? Do you still regularly use technology now that you can regularly visit in person?
- Can you describe what this experience (using technology) was like for you?
- Can you describe what if any type of help you received from staff members in the home to help you speak to your family using smart phones or tablets.
- In your experience can you describe how prepared family members and older adults are/were to socially connect with one another using technology.
- Can you describe what sort of supports you would like or would have liked to receive from the LTC?
- Did you receive help or support in using technology from any other person? *Prompt*: family member or friend, staff from LTC
- Can you describe what type of technology you use or used to connect with the older adult living in LTC.
- Can you tell me a little bit about your experience in using these types of technology for social connection. *Prompts*: is this something you do regularly? What challenges did you experience?
- Have you received any training or received any help in using technology such as smart phones or tablets? If yes from whom?
- Has your attitude towards using technology like smart phones and tablets changed since the COVID-19 pandemic? If so can you describe how?
- Are you aware of any programs or activities that may help you to use these technologies?
- Can you describe if and how using technology such as smart phones and tablets helped you feel more socially connected with your older adult family member living in LTC when you couldn’t visit in person.
- Can you describe some challenges or difficulties you experienced when using technology such as smart phones or tablets to connect with your family.

***Next Steps***

- Going forwards what would you like to see to more use of technology to support connecting with family members?
- Can you describe if and how you would like to see technology used to support care making decisions for older adults living in long-term care.
- Can you explain what would help you to use technology for the purposes of social connection.
- Can you describe what the LTC could do to help you use technology for social connection.
- Do you think you will use technology more going forwards? If yes or no, why or why not?
- Has your attitude about the use of technology to support social connectedness changed over the course of COVID-19 and if so how?
- From your perspective what do you think family members or older adults living in LTC need to help them socially connect with one another using technology such as smart phones and tablets?
- Were family members and older adults using these technologies to socially connect prior to COVID-19?
- Can you explain how you have supported family members in using technology to connect with an older adult living in LTC *prompts*: education, physically helping older adult etc.
- Has use of technology become normalized in LTC? Has this changed over the course of COVID-19?

***Next Steps***

- Going forwards what would you like to see to facilitate the use of technology for social connectedness within the LTC setting? *Prompts*: training, equipment, IT support
- Has your attitude about the use of technology to support social connectedness changed over the course of COVID-19 and if so how?
- Can you describe what is needed for optimal use of technology to support social connectedness within LTC.
- Do you see a role for both in-person visits and the use of technology like smart phones and tablets to connect with older adults living in long-term care and the home staff.
